# Supplementary material for: Structural snapshots of Xer recombination reveal activation by synaptic complex remodeling and DNA bending
Source: eLife. 2016 Dec 23;5:e19706. doi: 10.7554/eLife.19706 (PMC5241119; doi:10.7554/eLife.19706)
Supplement: Supplementary file 1. — DOI: http://dx.doi.org/10.7554/eLife.19706.022 [file elife-19706-supp1.docx]

**Supplementary File 1: List of oligonucleotides used in this study**

| **Oligonucleotide** | **Sequence** |
| --- | --- |
| **Crystallization of XerH synaptic complexes** | |
| *Pre-cleavage complex* | |
| difH-TS | GAGTTATGAAAACTGCACTTTTCAAACTTT |
| difH-BS | AAAGTTTGAAAAGTGCAGTTTTCATAACTA |
| *Post-cleavage complex* | |
| difHLP-TS | TAGTTATGAAAAC |
| difHLP-BS | TGCAGTTTTCATAACTA |
| **EMSA** | |
| difH-50-TS | TAAATTCATTTAGTTATGAAAACTGCACTTTTCAAACTTTTAAATCAAAC |
| difH-50-BS | GTTTGATTTAAAAGTTTGAAAAGTGCAGTTTTCATAACTAAATGAATTTA |
| difHL-TS | TAAATTCATTTAGTTATGAAAACTGCACTTAAATCAAACCATTTAAATAA |
| difHL-BS | TTATTTAAATGGTTTGATTTAAGTGCAGTTTTCATAACTAAATGAATTTA |
| difHR-TS | CATATTTTTCCTTTTAAATTCACTGCACTTTTCAAACTTTTAAATCAAAC |
| difHR-BS | GTTTGATTTAAAAGTTTGAAAAGTGCAGTGAATTTAAAAGGAAAAATATG |
| Random-TS | TATTATGTAAGTAACATAAGCTTATATGCGAAATAAACTGTTAAATAATC |
| Random-BS | GATTATTTAACAGTTTATTTCGCATATAAGCTTATGTTACTTACATAATA |
| **Analytical SEC** | |
| difH-30-TS | TAGTTATGAAAACTGCACTTTTCAAACTTT |
| difH-30-BS | AAAGTTTGAAAAGTGCAGTTTTCATAACTA |
| difHLP | TAGTTATGAAAACTGCAGTTTTCATAACTA |
| difHRP | AAAGTTTGAAAAGTGCACTTTTCAAACTTT |
| ***In vitro* cleavage assay with ‘suicide’ substrates** | |
| difH-nicked-TS-left | TTTAGTTATGAAAAC |
| difH-nicked-BS-left | TGCAGTTTTCATAACTAAA |
| difH-nicked-TS-right | TGCACTTTTCAAACTT |
| difH-nicked-BS-right | AAGTTTGAAAAG |
| Random-nicked-TS-left | TTTCCTTTTAAATTC |
| Random-nicked-BS-left | TGCAGAATTTAAAAGGAAA |
| Random-nicked-TS-right | TGCACTTAAATCAAAC |
| Random-nicked-BS-right | GTTTGATTTAAG |
| difH-unnicked-TS | TTTAGTTATGAAAACTGCACTTTTCAAACTT |
| difH-unnicked-BS | AAGTTTGAAAAGTGCAGTTTTCATAACTAAA |
| **Site-directed mutagenesis of XerH** | |
| SDM_Y344F | CAAGCCTGAATACCAGCCGTATTTTTACCCATTTCGATAAA |
| SDM_R213K | CTGCTGATTAAAATCATTGTGTTTACCGGTATGAAAAGCAATGAAGCACTGCAGCTG |
| SDM_K290S | GCACTGACCCAGGCATATCTGTATTCACAGGTTGAACGCATCATTAACTTTGC |
| **Amplification of *dif_H_* cassettes from *H. pylori* genome**  **(for DNaseI footprinting and construction of reporter plasmids)** | |
| difH-cassette-left-for | GATCGCTAGCGCAGATTGAAAAGGGGATTG |
| difH-cassette-left-rev | GATCTGTACATCTTTCTTGCGTTCTAAAATTGA |
| difH-cassette-right-for | GATCTCTAGAATTCGCAGATTGAAAAGGGGATTG |
| difH-cassette-right-rev | GATCGGTACCTCTTTCTTGCGTTCTAAAATTGA |
| **Site-directed mutagenesis of *dif_H_* cassettes in the reported plasmid** | |
| SDM-difHLP-left | CAAAGGGATAAAAAGAATTTTTATTTAAATGGTTTGATTTAAAAGTTATGAAAAGTGCAGTTTTCATAACTAAATGGGTAC |
| SDM-difHLP-right | CAAAGGGATAAAAAGAATTTTTATTTAAATGGTTTGATTTAAAAGTTATGAAAAGTGCAGTTTTCATAACTAAATGTGTACATTG |
| SDM-difHRP-left | GGTTTGATTTAAAAGTTTGAAAAGTGCAGTTTTCAAACTAAATGTGTACATTGCATGCATCGATAGATC |
| SDM-difHRP-right | GGTTTGATTTAAAAGTTTGAAAAGTGCAGTTTTCAAACTAAATGGGTACCATGGCGGTG |
| SDM-GT/CA-left | GGTTTGATTTAAAAGTTTGAAAAGTGCAGTTTTCATATGTAAATGTGTACATTGCATGCATCGATAGATCC |
| SDM-GT/CA-right | GGTTTGATTTAAAAGTTTGAAAAGTGCAGTTTTCATATGTAAATGGGTACCATGGCGGTGAAC |
| SDM-GT/CC-left | GGTTTGATTTAAAAGTTTGAAAAGTGCAGTTTTCATAGGTAAATGTGTACATTGCATGCATCGATAGATCC |
| SDM-GT/CC-right | GGTTTGATTTAAAAGTTTGAAAAGTGCAGTTTTCATAGGTAAATGGGTACCATGGCGGTGAAC |
| SDM-A/C-left | GGTTTGATTTAAAAGTTTGAAAAGTGCAGTTTTCAGAACTAAATGTGTACATTGCATGCATCGATAGATC |
| SDM-A/C-right | GGTTTGATTTAAAAGTTTGAAAAGTGCAGTTTTCAGAACTAAATGGGTACCATGGCGGTG |
| SDM-A/T-left | GGTTTGATTTAAAAGTTTGAAAAGTGCAGTTTTCAAAACTAAATGTGTACATTGCATGCATCGATAGATC |
| SDM-A/T-right | GGTTTGATTTAAAAGTTTGAAAAGTGCAGTTTTCAAAACTAAATGGGTACCATGGCGGTG |
| SDM-A/G-left | GGTTTGATTTAAAAGTTTGAAAAGTGCAGTTTTCACAACTAAATGTGTACATTGCATGCATCGATAGATC |
| SDM-A/G-right | GGTTTGATTTAAAAGTTTGAAAAGTGCAGTTTTCACAACTAAATGGGTACCATGGCGGTG |
